# Pathophysiologic Basis of Connected Health Systems

**DOI:** 10.2196/42405

**Published:** 2023-09-21

**Authors:** Yahya Shaikh, Michael Christopher Gibbons

**Affiliations:** 1 The MITRE Corporation Windsor Mill, MD United States; 2 The Greystone Group, Inc Lanham, MD United States

**Keywords:** smart health, connected health, systematic methodology, pathophysiology, architecting connected health systems, design, community, clinic, environment, system, technology, digital therapeutic, therapeutic systems

## Abstract

Since the start of the COVID-19 pandemic, there has been a rapid transition to telehealth across the United States, primarily involving virtual clinic visits. Additionally, the proliferation of consumer technologies related to health reveals that for many people health and care in the contemporary world extends beyond the boundaries of a clinical interaction and includes sensors and devices that facilitate health in personal environments. The ideal connected environment is networked and intelligent, personalized to promote health and prevent disease. The combination of sensors, devices, and intelligence constitutes a connected health system around an individual that is optimized to improve and maintain health, deliver care, and predict and reduce risk of illness. Just as modern medicine uses the pathophysiology of disease as a framework for the basis of pharmacologic therapy, a similar clinically reasoned approach can be taken to organize and architect technological elements into therapeutic systems. In this work, we introduce a systematic methodology for the design of connected health systems grounded in the pathophysiologic basis of disease. As the digital landscape expands with the ubiquity of health devices, it is pivotal to enable technology-agnostic clinical reasoning to guide the integration of technological innovations into systems of health and care delivery that extend beyond the boundaries of a clinical interaction. Applying clinical reasoning in a repeatable and systematic way to organizing technology into therapeutic systems can yield potential benefits including expanding the study of digital therapeutics from individual devices to networked technologies as therapeutic interventions; empowering physicians who are not technological experts to still play a significant role in using clinical reasoning for architecting therapeutic networks of sensors and devices; and developing platforms to catalog and share combinations of technologies that can form therapeutic networks and connected health systems.

## Introduction

Since the start of the COVID-19 pandemic, there has been a rapid transition to and uptake of telehealth across the United States, primarily involving virtual clinic visits [1]. The promise of telehealth, however, involves more than virtual visits to a clinician and includes sensors and devices surrounding a person in their home and community to facilitate health and well-being [2-10]. An ideal state of telehealth is one where environments in which people are born, live, play, work, and grow old are networked and intelligent and automatically promote health and prevent disease in a way that is personalized to each individual within them [11]. These personalized connected health systems surrounding each individual can, for example, in persons with Crohn disease, serve to predict and reduce risk of a flare-up by monitoring the presence of biomarkers or changes in gut flora by sampling fecal matter in the toilet; in persons with ADHD, autism, or anxiety disorders, these system can use image analysis and wearables to detect mood and agitation and responsively modulate the environment to prevent the onset of acute symptoms. Expanding the deployment of telehealth to include systems of purposefully connected sensors, devices, and machine intelligence—connected health systems—surrounding individuals requires an approach that is systematic and based in clinical reasoning. Such an approach can potentially lead to disruptive innovation in health, including smart homes and work places optimized for the health of their residents; prescriptions for connected health environments; and reimbursement by payers for deploying connected health systems around their beneficiaries to improve health while reducing cost. In this paper we propose a methodology to engineer connected health systems around patients that is rooted in the pathophysiology of disease. Our intention is to propose a framework that can be used to identify how consumer and health IT can be structured around a patient to optimize for health. This approach provides a tool for innovators to identify pain points and opportunities in constructing ecosystems for health using foundational clinical reasoning. We apply the approach to a hypothetical patient with diabetes and discuss the significance of the methodology to health in an increasingly networked world.

## Starting With the End in Mind: Using Intervention Levels and Practice Levels to Define a Connected Health System’s Targets for Therapy and Optimization Space

We propose combining a person-centered design with physiologic and pathophysiologic mapping of health and disease as the foundation for designing connected health systems. The design of a connected health system around individuals will be influenced by the intended levels of intervention and the desired practice level of the system. The intended levels of intervention are the primary (mitigating risk of disease), secondary (interrupting disease process before clinical manifestation), or tertiary (mitigating complications from disease) levels of health prevention that the system is intended to impact as part of its optimal function [12,13]. The practice level consists of the extent to which clinicians and health care workers will expand the connected health system’s targets for therapy beyond the biological elements of a person’s health and illness to also consider environmental, social, community, and other aspects impacting health and well-being [14-18]. At each practice level, a different set of system outputs would be expected based on the intended intervention level. The choice of the intended level of intervention and practice level should be informed by a number of practical considerations that need to be taken into account by those implementing the connected health system around individuals. These considerations include, but are not limited to, the availability of resources for initiating and maintaining the system at the particular practice level, the consent of individuals around whom such systems are implemented, the availability of system components for initial construction and subsequent maintenance and replacement if needed, the availability of technical expertise to interface components and troubleshoot, and the availability of connectivity infrastructure necessary to support the deployment of the connected health system around the individual. It is important to give thought to identifying the intended levels of intervention and practice levels since choices surrounding them will lead to a different optimization space, structure, function, and emergent properties of the connected health system.

## Evidence-Based, Person-Centered Design: A Systematic Methodology to Design Connected Health Systems

After identifying the intended levels of intervention and practice level of the system, the following 5 steps guide the design of a connected health system around an individual that is person-centered and rooted in evidence-based physiologic and pathophysiologic models of health and disease.

### Step 1: Develop Personas That Represent the Lived Experience of the Individual

This initial step serves to characterize the biological and social determinants surrounding the person while identifying pathologies for which they are at risk or for which they already have a diagnosis. This helps represent the health experience and profile of a person and can give an idea of how a connected health system around the individual would impact their life.

### Step 2: Map Initial Pathophysiologic Pathways

From the persona developed in step 1, we identify the health status of interest and relevant conditions as targets for sensing and intervention. This information is used to develop a map of the pathophysiologic pathways of the patient’s health and conditions. Cause-effect maps of normal and disrupted physiology are defined and available in literature at varying levels of details [19-25]. The clinician’s task is to integrate various maps together at an appropriate level of detail up to the intended practice level and for the intended levels of intervention. This integrated map then serves as the skeleton around which a system of connected data inputs, signal outputs (eg, to connected devices), and connected intelligence can be architected and networked.

### Step 3: Expand the Pathophysiologic Map to Include Determinants

This step expands the initial pathophysiologic pathway characterized above to include social and economic levels to the extent necessary to be inclusive of the practice level identified as part of the early design activities.

### Step 4: Identify Targets for Sensing and Intervening

Using the maps developed above, identify targets for using connected sensors to collect data or connected devices to intervene to achieve a desired outcome. This step defines the particular sensors and devices that can be used for each target. Connected data inputs include connected sensors that collect data about an individual’s physiology and their physical and social environments. Data inputs can also include surveys collected about an individual and their communities, real-time data from user response, and population-based historic data that can inform predictive and classification algorithms.

### Step 5: Connect System of Sensors and Devices Through Tailored Machine Intelligence

In order to construct a network of sensors and devices in a way that can represent a system optimized to improve the health of individuals, intelligence is needed to process data from sensors (inputs) and output signals that can serve multiple purposes including controlling relevant devices to achieve an intended impact. Signal outputs (such as to the connected devices) emerge from machine intelligence that connects data inputs to some action. These actions can include, but are not limited to, further data inputs to the connected system, messages to individuals or other communicating systems (eg, electronic health records), and modification of connected devices in order to modulate the environment around the individual. Connected intelligence is the networked analytic stack that modulates the relationship between the person and the connected data inputs, the relationship between groups of sensors, the relationship between groups of devices, and the relationships between connected inputs and signal outputs in order to achieve the outcome at the intended levels of intervention for the given practice levels identified earlier.

## Applying the Clinically-Oriented, Evidence-Based, Person-Centered Design Methodology as a Systematic Way to Design Connected Health Systems: Notional Example

The following example demonstrates the application of the clinically-oriented, evidence-based, person-centered design (CORE-PCD) methodology toward designing a connected health system.

### Develop Personas That Represent the Lived Experience of the Individual

A 50-year-old man has a history of obesity and diabetes initially diagnosed 1.5 years ago, and currently being managed with metformin and glipizide. His family history is significant for a father who had chronic kidney disease secondary to uncontrolled hypertension and died from a stroke when he was 55 years old. His mother is 95 years old and is currently living with him. She has mild arthritis in her hands and knees, but is otherwise healthy, active, and independent, performing all activities of daily living and instrumental activities of daily living without assistance. The man lives in a large city in an apartment building facing the main street with considerable vehicular traffic and often with sounds of police sirens and helicopters. The street has a high risk of crime, and he finds himself unable to walk or be physically active outside the home in the way he would want. He works at a call center where he is often seated at a desk and reports his work environment as being stressful because of monthly downsizing as the company grapples with a downturning market. Regular downsizing has been ongoing over the last year and has led to an increasing burden of work on current employees without additional pay or hours. On most days, he eats lunch and dinner from the food truck outside his work or from a fast-food restaurant. He has also been complaining of poor sleep quality. The pressure to perform at work has prevented him from asking for time off to keep his regular clinic appointments, and he has missed his last 2 appointments this year. At his last clinic visit, about a week ago, he presented with polyuria, nocturia, thirst, and fatigue. A physical exam was significant for an obese male with a muted affect. Lab results, which are available for his visit today, are significant for a glycated hemoglobin level of 10.5 mg/dL, and in today’s visit he is started on long-acting insulin with premeal insulins added to normalize postprandial blood glucose; he is started on low-dose aspirin and a statin to reduce his risk of atherosclerotic cardiovascular disease; and he is offered counseling on life style modification, including physical exercise, diet, and nutrition.

### Map Initial Pathophysiologic Pathways

**Figure 1:**
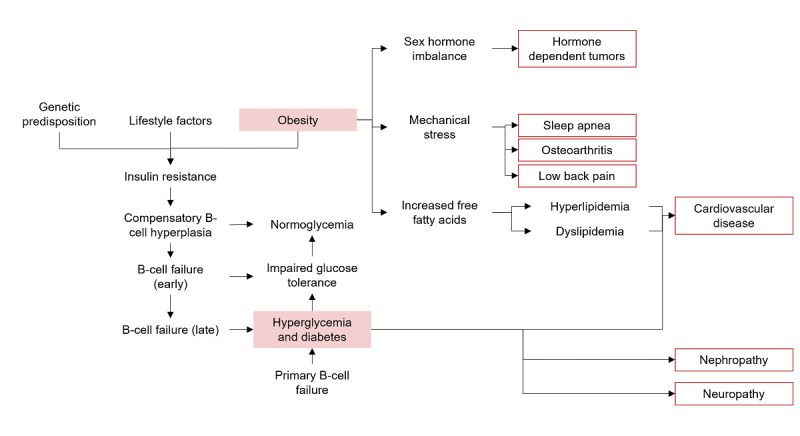
Pathophysiologic map.

In this step we map the diabetes and obesity pathways below for our example patient (from the persona in step 1 above). The biochemical detail for pathways can vary depending on the purpose of the mapping: if it is done for innovation and identifying the targets for sensing and intervention, a more detailed biochemical conceptualization may be warranted. For the purpose of deployment of a connected environment around a patient to manage their condition, a more abbreviated version can suffice based on what is technologically available at that time (Figure 1). For illustrative purposes, we characterize a more detailed version for the diabetes diagnosis and an abbreviated version for obesity that is focused on managing sequelae.

### Expand the Pathophysiologic Map to Include Determinants

**Figure 2:**
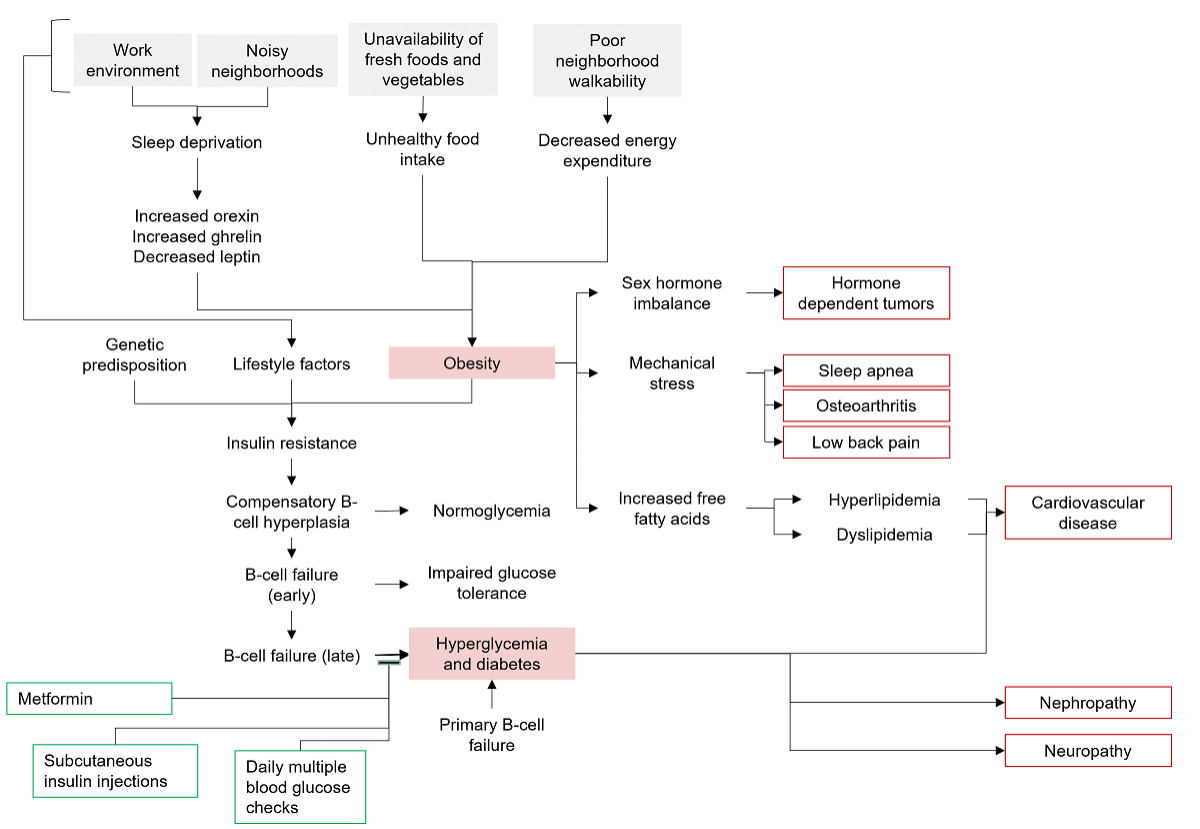
Expanded pathophysiologic map.

In this step we expand the pathophysiologic pathway in terms of sociologic and economic levels to help identify predetermining factors of pathologies that lie in lifestyle, behavioral, and social aspects of a person’s life (Figure 2). Pharmacologic interventions can be included as part of the clinical profile of the patient. If the patient has multiple pathologies, they may be related together through factors that are common between their pathophysiologic pathways.

### Identify Targets for Sensing and Intervening

**Figure 3:**
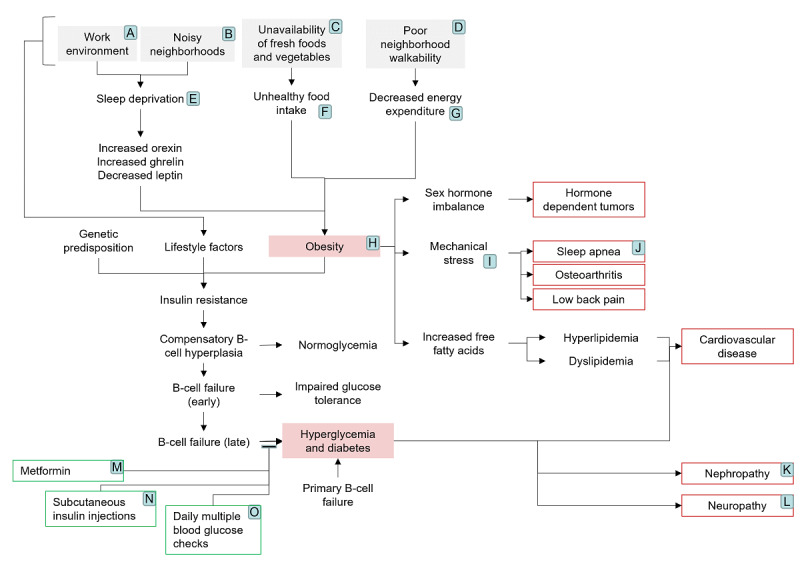
Targets for sensing and intervening. A: Work stress data sets; B: environmental noise sensors; C: food environment data sets (eg, food desert status and fast-food scores); D: real-time traffic data sets, real-time crime reports, and walkability scores; E: sleep quality sensors; F: food intake trackers and electronic food diaries; G: fitness trackers; H: connected scales and body fat percentage trackers; I: connected insoles, posture detectors, mechanical stress sensors, and sleep apnea detectors; J: apnea monitoring; K: connected protein sensors that sample material in the toilet; L: activity monitoring devices that can help detect and monitor progression of loss of sensation or perform surveys to measure the level of neuropathic pain; M: connected medicine bottle or smart cap; N: connected syringe and connected insulin bottle; O: continuous glucose monitoring.

In the flowchart visualization of the completed pathway, we are able to identify nodes as opportunities for deployment of connected sensors and devices as targets of interventions (Figure 3). Based on these nodes we are able to catalogue technologies that may be available for deployment of connected environments for patient management. Interventions at nodes that are at the nexus of multiple pathways can be valuable in addressing multiple conditions. In the illustration below, those factors that currently have an associated technology available on the market are identified by a letter. Each letter corresponds to a catalogue of technologies. Combinations of these can be deployed around an individual as needed to construct combinations of sensing and intervening technologies that form connected environments personalized for that individual.

### Connect System of Sensors and Devices Through Tailored Machine Intelligence

**Figure 4:**
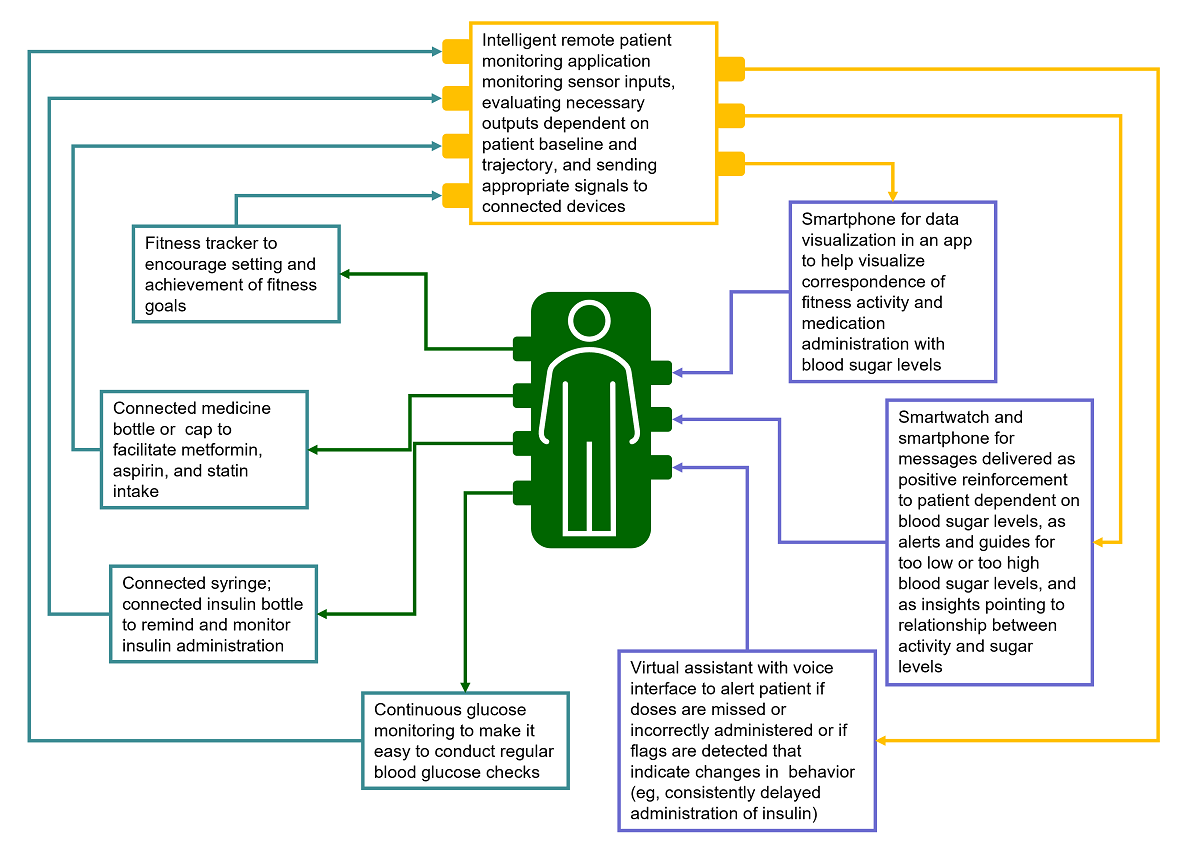
Initial connected system deployed around patient.

Data collected from a node are informative for care delivery and intervention. Between data collection (eg, through a connected sensor) and intervention, the data has to be processed and integrated into an algorithm for intelligent responses: the afferent sensor data are input into machine intelligence which output an efferent signal. Machine intelligence algorithms can become personalized to the particular needs of an individual where a person is their own baseline. Automatic, ongoing assessment of a person through sensors, questionnaires, and advanced analytics can allow better characterization of evolving profiles of a person and their environment. In addition to helping manage current disease states, it can help with prediction of risk factors and additional disease states for which the person may be susceptible over time as they progress through life. In the case of the patient above, the physician decides to initiate a connected ecosystem to support care delivery involving sensors (fitness tracker, connected medicine bottle, and connected syringe), devices (smartphone, smartwatch, and virtual assistant), and artificial intelligence (AI) in the form of a remote patient management application (Figure 4).

In future visits the physician in the above example can plan to extend the initial connected health system around the patient to include a connected scale, food environment data sets relevant to the patient’s work and home environments, and sleep quality sensors. The provider can additionally revisit the patient’s community-generated data, reveal insights to the patient in relation to their health, and use these to inform medication adjustments, if necessary (eg, modify the insulin dosing algorithm to match the patient’s activity level in a way that prevents hypoglycemia). The provider can use a health care ecosystem management interface to set appropriate thresholds (eg, glucose above or below a threshold) to send an appropriate signal (eg, to the connected device, patient’s chart, caregiver, or the patient themselves as a message).

## Using Clinical Reasoning to Design Connected Health Systems: New Opportunities for Providers in the Information Age

We present above a systematic methodology for clinicians and innovators to organize a large volume of available technologies into deployments that are rooted in the pathophysiologic basis of disease. The above methodology can be applied to specific disease processes and health states while being technology agnostic. It can be used by clinicians to personalize digital health ecosystems from first principles rooted in clinical reasoning.

Connected technologies are now ubiquitous and relevant in most facets of our lives, from accessing social determinants of health to gaining health care delivery. It is becoming increasingly important for there to be a pathway to formally train clinicians or other dedicated staff to gain domain-specific knowledge in constructing connected health systems [26].

The method presented in this paper can be of benefit to clinicians who do not have deep technical knowledge of IT but want to participate in shaping technological innovation. Physicians can combine their understanding of pathophysiology and natural history of disease with the expertise of a technical start-up to advise entrepreneurs on how to visualize and construct ecosystems of health. Technology entrepreneurs who may not have a background in health may also find it appealing to connect with providers who have an ability to use their understanding of health and disease in a systematic clinical-reasoning framework to construct technology solutions.

The above thought process may additionally become important to the practice of medicine, as devices that can impact health proliferate in the consumer and regulated health spaces. However, it is not necessary for every provider to be an expert in health IT. In the current practice of medicine, a clinician needs to have sufficient understanding of the pathophysiologic basis of pharmacologic therapy to appropriately prescribe combinations of therapy to match the peculiarities of an individual patient. However, with some exceptions, in most cases it is not clinically relevant to know how the bioassays and mouse models were generated for the medication being prescribed. Similarly, for clinicians to construct a connected health ecosystem for their patients, a key competency is needed: using developed devices and apps already approved for clinical use. Not every physician needs to have a developer-level knowledge of devices and applications. However, every physician in the information age can benefit from a rationale of how to put various approved devices and applications together in a way that can improve the health of their patients.

While it may be important for clinicians to know how to construct connected health ecosystems, given the current demands of the clinical setting it cannot be expected that all clinicians would have an equal level of competency. However, a role for clinical informaticists may need to be more prominent, perhaps even as a specialist to whom referrals can be made in order to recommend an optimally beneficial, connected health ecosystem for a given patient.

This paper describes the application of a pathophysiologic thought process to conceptually structure the space of health IT around a patient living in the community. This is beneficial for innovators who can use the approach presented above to identify pain points and opportunities for innovation and entrepreneurship: What standards might be necessary for devices to communicate in an ecosystem? How might AI or machine learning models take into account real-time data generation to inform their outputs? How might analytic algorithms use large data sets and population level data to train a model while ensuring the patient retains their own control for personalized care? How might an ecosystem for health be implemented while ensuring privacy and security? In attempting to answer these questions, innovators can look to other industries that have also implemented ecosystems of devices and intelligence. Smart buildings and smart homes are examples of industries that have preceded the health industry in formulating smart environments. Instead of architecting smart homes that optimize energy or cost, smart environments can additionally optimize health.

Additionally, advances in domains such as connected networks (eg, 5G and 6G), advanced computing (eg, advanced AI or machine learning models), sensors (eg, devices that measure physiologic data in the community), interfaces (eg, between user and device or between devices), and material sciences (eg, for maintaining Moore’s law or developing new types of biologically interfaced connected sensors and devices) suggest that connected health in the community, beyond the walls of the clinic, is an emerging health system that is decentralized and driven by consumer needs and expectations [11]. This will necessitate clinical medicine and public health to take the initiative in ensuring that recommendations for applying interconnected systems of technologies to patient care and well-being happen in a way that is rooted in clinical reasoning and applied though evidence-based practice.

Directions for future research can clarify the interaction between classes of technologies and types of deployments necessary for optimal care delivery. For example, for the class relevant to person-technology interface, clarity is needed around health states and optimal interface (eg, process for developing personalization and predictiveness; extent of interactivity versus passivity; considerations in embedding sensors, devices, and intelligence into living and community spaces, environments, and objects; and alternate interfaces such as brain-computer, tactile, haptic, gesture, immersive, or voice). Similarly, other classes of technologies necessary for functional smart ecosystems of health need to be better understood.

In summary, it is feasible to construct connected health systems using a methodology rooted in the pathophysiologic basis of disease. This is aligned with the type of clinical reasoning that is familiar to clinicians [27,28] and can provide a systematic and repeatable methodology for the development of care pathways, evidence-based guidelines, and measurable quality metrics for deploying smart ecosystems and environments for health around individuals and communities.

## Abbreviations

AI: artificial intelligence
CORE-PCD: clinically-oriented, evidence-based, person-centered design
